# Full Mitochondrial Genomes Reveal Species Differences between the Venerid Clams *Ruditapes philippinarum* and *R. variegatus*

**DOI:** 10.3390/genes13112157

**Published:** 2022-11-19

**Authors:** Yumei Tang, Zhongming Huo, Yang Liu, Yuhang Wang, Luya Zuo, Lei Fang, Wen Zhao, Yue Tan, Xiwu Yan

**Affiliations:** 1 College of Fisheries and Life Science, Dalian Ocean University, Dalian 116023, China; 2 Engineering Research Center of Shellfish Culture and Breeding in Liaoning Province, Dalian 116023, China; 3 School of Marine Science and Environmental Technology, Dalian Ocean University, Dalian 116023, China

**Keywords:** *Ruditapes philippinarum*, *Ruditapes variegatus*, mitochondria, mitogenome, Veneridae

## Abstract

In natural sea areas along the coast of China, venerid clams *Ruditapes philippinarum* and *R. variegatus* exhibit similar adult shell forms and are especially difficult to distinguish as spat and juveniles. This study used comparative mitochondrial genome analysis to reveal differences between these species. The results showed that: (1) the mitochondrial genomes of *R. philippinarum* and *R. variegatus* share a large number of similar gene clusters arranged in consistent order, yet they also display noncommon genes, with both gene rearrangements and random losses found; (2) the 13 protein-coding genes in *R. philippinarum* as well as two-fold and four-fold degenerate sites in *R. variegatus* have an evident AT bias; (3) the Ka/Ks ratio of the mitochondrial *ATP8* gene was significantly higher in *R. philippinarum* than in *R. variegatus*, and an analysis of selection pressure revealed that the mitochondrial NADH dehydrogenase subunit 2 gene and NADH dehydrogenase subunit 6 gene of *R. variegatus* were under great selective pressure during its evolution; and finally, (4) the two species clustered into one branch on a phylogenetic tree, further affirming their phylogenetic closeness. Based on these results, we speculate that the species differences between *R. variegatus* and *R. philippinarum* are largely attributable to adaptive evolution to the environment. The present findings provide a reference for the development of germplasm identification.

## 1. Introduction

**Figure 1:**
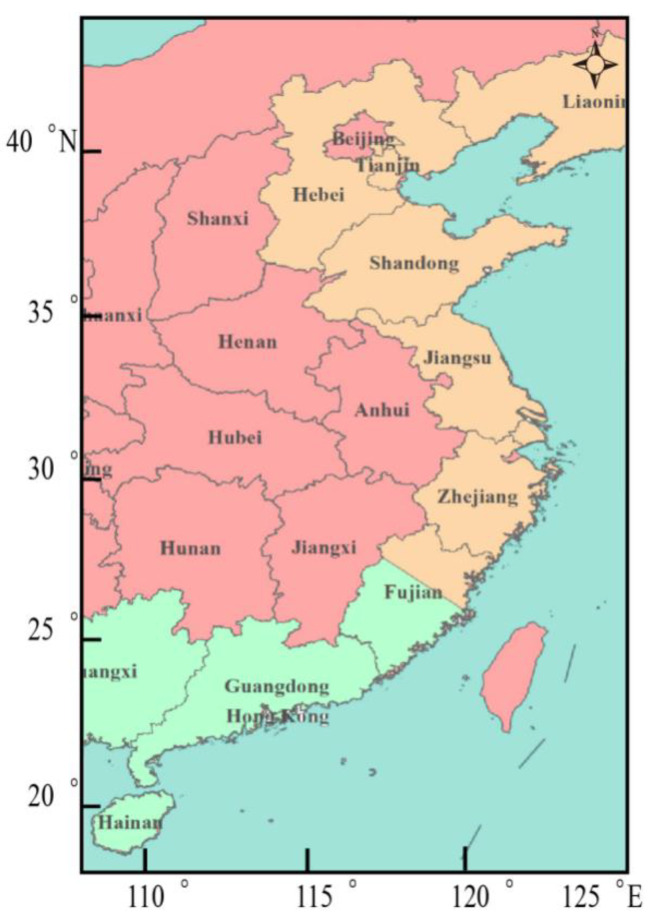
Coastal provinces of China where *Ruditapes philippinarum* (orange and green) and *R. variegatus* (only green) are found in natural seawater areas.

The economically important Manilla clam *Ruditapes philippinarum* has a wide distribution in coastal waters of the Indo-Pacific, including along the northern and southern coasts of China, such as the Liaoning, Hebei, Guangdong, and Fujian provinces. The species is cultured worldwide, and its annual production exceeds 3 million tons in China, where it is one of the four most farmed bivalve species and is the highest yielding, accounting for 22% of the output of shellfish culture [1]. The variegated carpet shell *Ruditapes variegatus* likewise has an Indo-Pacific distribution, but with less natural production; in China it occurs mainly along the south coast, notably in Pingtan County in Fujian Province [1] (Figure 1). Both species are found in natural seawater areas in Guangdong Province, and they are difficult to distinguish based on the external appearance of their shell, as well as at the spat and juvenile stages. Therefore, these species require study through the help of molecular markers to clarify whether this phenomenon is a result of gene introgression or natural hybrid progeny.

Mitochondrial DNA (mtDNA) is widely used in aquatic animal classification and species identification. Several studies have demonstrated that mitochondria can simultaneously regulate the death of cells and sense and respond to extracellular and intracellular signals and pressures [2,3,4,5,6,7,8,9,10,11]. Mitochondria are the ‘power station’ of the cell and provide energy through peroxidative phosphorylation, as well as the capacity for aerobic respiration in cells. Mitochondria are able to regulate cell death, act as energy sensors, and can respond to extracellular and intracellular signals and stresses. Most animal mtDNA is a closed circular double-stranded DNA molecule consisting of 13 protein-coding genes (PCGs), 22 tRNA genes, two rRNA genes, and one or two non-coding regions (Boore, 1999). mtDNA is characterized by small molecular weight, high energy content, simple structure, fast evolution, and maternal inheritance [12,13], highly conservative gene sequences [12], and high variability of gene order [14].

To date, reports on the mitochondrial genomes of aquatic animals have focused mainly on: (1) phylogenetic analyses, including relationship reconstructions; (2) between-species comparisons, such as through analysis of conserved gene clusters and determinations of the evolutionary conservation of species; (3) the phenomenon of gene rearrangements; (4) the mechanisms of tandem duplications; and (5) analyses of evolutionary selection pressures [14,15,16,17,18,19,20,21,22,23,24,25]. For instance, Tang et al. confirmed the status of the crab *Helicana wuana* (in the family Varunidae of subsection Thoracotremata) by analyzing the basic characteristics, gene arrangement, and phylogenetic inference of the mitochondrial genes [12]. Liu et al. compared structural characteristics of the mitochondrial genome and gene sequences among 16 bivalve species and found that the mitochondrial gene sequences were not obvious and their structures varied [26]. Iannelli et al. confirmed interspecific relationships in ascids by comparing mitochondrial genomes [13]. Sun et al. analyzed complete mitochondria from two species of Arcidae, *Trisidos kiyoni* and *Potiarca pilula*, and identified complex gene rearrangements in the mitochondrial genome as a key character in inferring the higher-level phylogenetic relationship of the Arcidae [27]. A recent study has shown that *Ruditapes philippinarum* and *R. variegatus* may belong to different genera, but a fully revised classification will require considerable additional genomic and morphological data from a wide sample of species and genera (Liu et al., 2022). In this study, we conducted a comparative analysis of the mitochondrial genomes of the two species to better differentiate them. This included assembly of the whole mitochondrial genome, as well as analyses of the gene content, repetitive sequences, gene arrangements, codon selection preferences, and selection pressure. Comparative analyses to determine differences between two mitochondrial genomes in the process of species differentiation accompanied by phylogenetic analysis can provide a basis for molecular species identification and the protection of genetic diversity and shellfish germplasm genetic resources for aquaculture.

## 2. Materials and Methods

### 2.1. Material Collection

**Figure 2:**
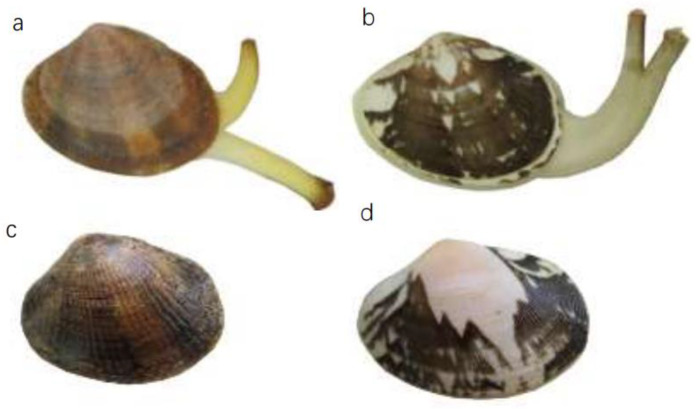
Comparison of the external radial ribs and degree of separation of the incurrent and excurrent siphons of (**a**,**c**) *Ruditapes variegatus* and (**b**,**d**) *R. philippinarum*.

Specimens of *R. philippinarum* and *R. variegatus* were collected in Zhanjiang, Guangdong Province, China. The collected animals were transported to the laboratory of the Liaoning Engineering Research Center of Dalian Ocean University. The clams were temporarily maintained for one week in seawater with a temperature of 17 °C, salinity of 30 ppt, and pH 7.76–8.5, and were fed *Chlorella*. During this time, the degree of separation between the clams’ incurrent and excurrent siphons was observed to facilitate sorting of the two species to ensure the accuracy of the experimental material. In *R. variegatus*, the tubes appear completely separated at the base (Figure 2a), whereas in *R. philippinarum* the tubes are separated a short distance from the distal end (Figure 2b). The radial ribs of *R. philippinarum* are raised, thin, and closely spaced, and number approximately 90–100 (Figure 2c), while those of *R. variegatus* are more flattened and number approximately 50–70 (Figure 2d). Measures of shell length, shell width, shell height, soft-tissue dry weight, and hypertrophy in *R. variegatus* and *R. philippinarum* are given in Table 1. The values of the measured phenotypic traits did not differ significantly between species (*p* < 0.05).

### 2.2. Mitochondrial Genome Sequencing, Assembly, and Analysis

#### 2.2.1. PCR Amplification and Sequencing

One specimen each of *R. variegatus* and *R. philippinarum* was selected as the experimental sample for total DNA extraction (using tissue of the dissected foot) with a Marine Animals DNA Extraction Kit (Shenzhen Huitong Biotechnology Co., Ltd., Shenzhen, China). The PCR amplification system was a 10-µL La-Taq system with the following reaction conditions: 0.1 µL of La-Taq, 0.5 µL of template DNA, 0.5 µL of each the forward and reverse primers, 1 µL of buffer, 1.6 µL of dNTPs, and 5.8 µL of ddH_2_O. The specific reaction steps were initial denaturation for 5 min at 94 °C, followed by 35 cycles of 5 min denaturation at 94 °C, annealing for 30 s at 50 °C and extension for 1 min at 72 °C, with the three steps repeated 35 times, and the final extension step carried out at 72 °C for 7 min. The PCR products were detected by agarose gel electrophoresis. The PCR amplification products were used as the samples for mitochondrial genome sequencing.

Sequencing of the total DNA sample was performed with Illumina next-generation sequencing technology. After build the Illumina paired-end DNA libraries, the raw image data obtained from Illumina HiSeq sequencing were transformed into sequence data after base calling. Raw sequencing data was uploaded to the NCBI (*Ruditapes philippinarum* Raw sequence reads, PRJNA896365; *Ruditapes variegatus* Raw sequence reads, PRJNA896706). Next, paired-end sequencing of the sample DNA used Illumina HiSeq sequencing to make the subsequent assembly more accurate, with shearing of the low-quality data in the raw sequencing data. Next, quality control (QC) of the sequencing data was performed with NGS QC software; the QC scores did not indicate low-quality read lengths.

#### 2.2.2. Sequence Splicing and Gene Annotations

Gene sequence splicing was preliminary clean data on QC using SPAdes 3.9.0 to splice out all the scaffold that could be spelled out in the clean data. Next, blastn and exonerate alignment were performed using published data on the near-source organelle genome and sequences of the protein-encoding genes (PCGs) as a reference. The matching scaffold genes were then picked out; the coverage obtained after splicing was also ordered, and fragments that were clearly not the target genome were removed. The collected fragmented target sequences were used to extend and merge the splicing with PRICE and MITObim. The results of iterative splicing were data mapped against the original sequencing read lengths using bowtie2, and pairwise reads on the match were then singled out and restitched using the software SPAdes. Finally, the formed circular genome was extracted.

Genome annotations include the annotation of PCGs, RNA annotation, and structural annotation. PCG annotation was performed to confirm gene sequences and boundaries through blastn alignment of protein and protein-coding sequences from near-source species. The UGENE Open Reading Frames (ORFs) finder was then used. The Invertebrate Mitochondrial Code table was selected for sequence ORF prediction, and the predicted ORF was aligned using blastp to the nr database to annotate its function. The rRNA annotations were submitted to the RNAmmer 1.2 server (http://www.cbs.dtu.dk/services/RNAmmer, accessed on 15 November 2022.) for sequence prediction, supplemented by an orthologous sequence alignment, to correct for the boundary range. After sequence annotation, the Sequin software tool was used for structure annotation to submit the edited file to OGDRAW, which converts annotations in the GenBank format to graphical maps.

#### 2.2.3. Analysis of Selective Pressure on the PCGs

To reflect the selective pressure on the PCGs of *R. philippinarum* versus *R. variegatus* during natural evolution, we selected two other venerid species, *Antigona lamellaris* and *Paratapes textilis*, as reference species based on the results of phylogenetic analysis. The advantage is that comparative analysis of selection pressure using species that differ in proximity and distance allows a more accurate search for PCGs in two similar species in which mitochondrial genetic variation rapidly aggregates. We calculated the ratio of the nonsynonymous substitution rate (Ka) to the synonymous substitution rate (Ks) of each PCG in *R. philippinarum*, *R. variegatus*, *A. lamellaris*, and *P. textilis* (i.e., *R. philippinarum* vs. *R. variegatus*; *R. philippinarum* vs. *A. lamellaris*; *R. philippinarum* vs. *P. textilis*; *R. variegatus* vs. *R. philippinarum*; *R. variegatus* vs. *A. lamellaris*; and *R. variegatus* vs. *P. textilis*). We speculated that in the natural evolution of *R. philippinarum* and *R. variegatus*, natural selection will act on the orientation and size of the PCGs in the target species. Sequences were aligned and formatted using the default parameters of ClustalW 2.1 and ParaAT2.0, respectively. The values of Ka, Ks, and the Ka/Ks ratio were calculated with the YN method of the KaKs_Calculator 2.0 software package (wangdp, Britain (UK)/GMT, https://sourceforge.net/projects/kakscalculator2/, accessed on 15 November 2022); the invertebrate mitochondrial code was selected as the reference genetic codes; and Fisher’s exact tests were performed to verify the validity of the Ka and Ks values [28,29].

#### 2.2.4. Phylogenetic Analysis

To accurately determine the position of *R. philippinarum* in a phylogeny with *R. variegatus*, the whole-genome nucleotide sequences of 24 mollusk species were downloaded from GenBank (https://www.ncbi.nlm, accessed on 12 October 2021). These included species of Vesicomyidae (4 species), Mactridae (4 species), Veneridae (14 species), Sepiidae (1 species), and Ostreidae (1 species), with the Sepiidae and Ostreidae representing exogenous populations. Gene-length information and GenBank accession numbers by species are listed in Table 2. The whole-genome length of the mitochondrial DNA is approximately 15,000–22,000 bp. A phylogenetic tree was constructed for *R. philippinarum* and *R. variegatus* using the maximum-likelihood method and the whole-mitochondrial DNA genome sequences of the 24 downloaded mollusk species, with a bootstrap test value set to 1000.

## 3. Results

### 3.1. Mitochondrial Genome Features of R. variegatus and R. philippinarum

**Figure 3:**
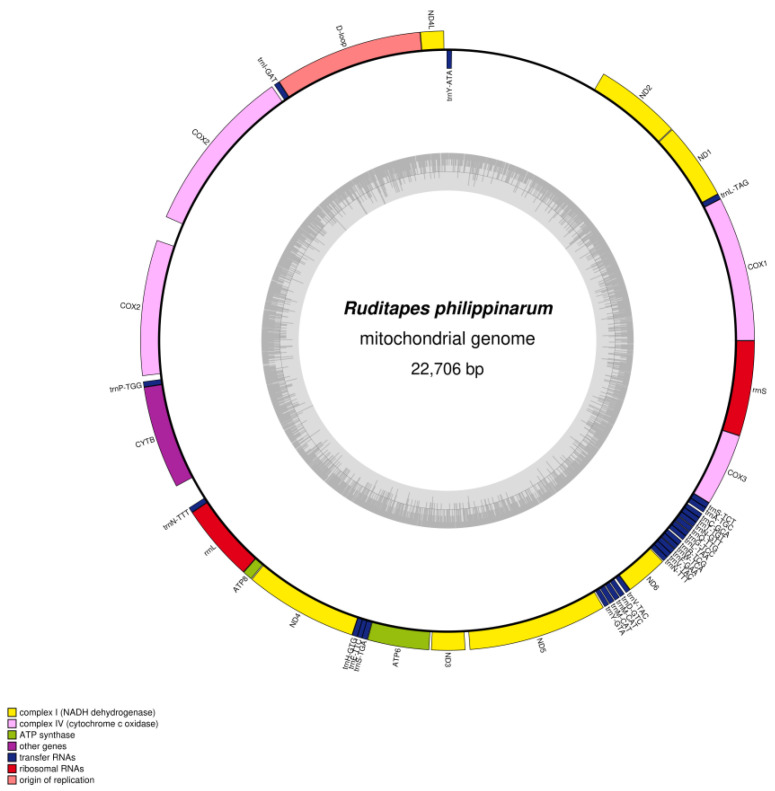
Circular genome map of *Ruditapes philippinarum* mitochondrial DNA. The transcription direction of the gene in the ring proceeds clockwise, while transcription of the gene outside the ring proceeds counterclockwise. Different functional genes are marked with different colors. The inner gray circle depicts GC content along the genome, and the gray line at the center denotes the 50% threshold.

**Figure 4:**
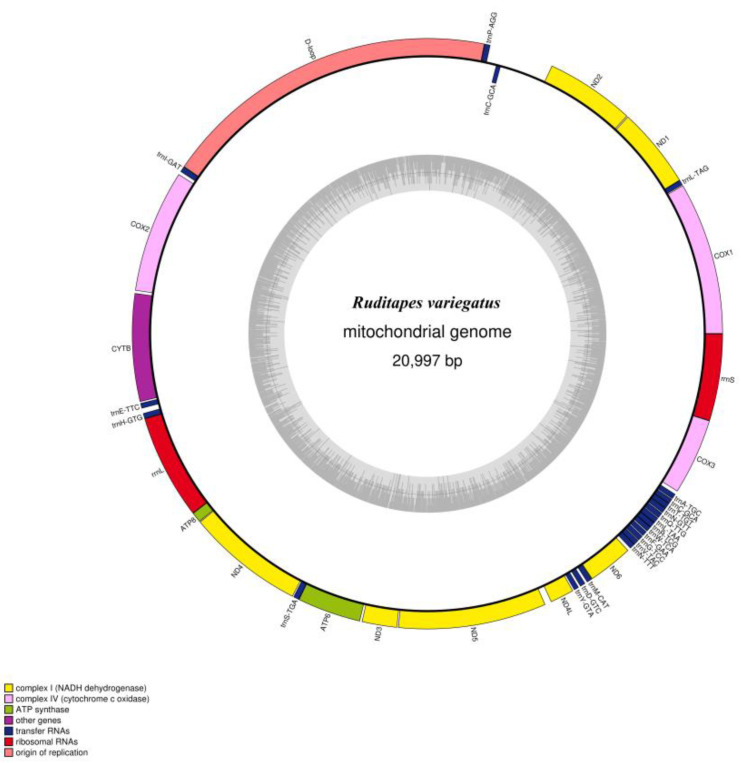
Circular genome map of *Ruditapes variegatus* mitochondrial DNA. The transcription direction of the gene depicted in the ring proceeds clockwise, while transcription of the gene outside the ring proceeds counterclockwise. Different functional genes are marked with different colors. The inner gray circle depicts GC content along the genome, and the gray line at the center denotes the 50% threshold.

We determined the whole mitochondrial genome sequences of *R. philippinarum* and *R. variegatus*. The nucleotide sequences were uploaded to GenBank (https://www.ncbi.nlm.nih.gov/genome, accessed on 15 November 2022), with accession numbers MZ675529 and MZ675530 for *R. philippinarum* and *R. variegatus*, respectively. The mitochondrial genome of *R. philippinarum* is 22,706 bp, and *R. variegatus* is 20,997 bp; circular maps of the mitochondrial whole genome structure and loop annotations are presented in Figure 3 and Figure 4.

The total genome-wide GC content of *R. philippinarum* was 30.3%, and the composition of the whole mitochondrial nucleotide genes was A 30.2%, C 9.8%, G 20.5%, and T 39.6%, with an AT bias. Similar to the metazoan mitochondrial genome, the whole mitochondrial genome in *R. philippinarum* consists of 42 unique coding genes, comprising 14 PCGs, 26 tRNA genes, 2 rRNA genes, and 1 D-loop control region (Table 3). Furthermore, the genes were found to contain duplicates of the *COX2*, *trnM-CAT*, *trnN-GTT*, and *trnV-TAC* genes. In *R. philippinarum* the total length of the PCGs was 14,454 bp, representing 63.66% of the entire mitochondrial genome; the D-loop region represented 7.83%; and the tRNA and rRNA contents were 7.42% and 9.05%, respectively (Table 4).

The total genome length of the mitochondria of *R. variegatus* was 20,997 bp, with a total GC content of 36.4%, and other nucleotide content of A 28.1%, C 10.7%, G 25.7%, and T 35.5% (Table 5). The whole mitochondrial genome of *R. variegatus* consists of 37 unique encoding genes, comprising 13 PCGs, 22 tRNA genes, and 2 rRNA genes. The D-loop region, consisting of 18.53% of the entire mitochondrial genome length and 3892 bp, is the starting point of replication (Table 6). Only the *trnC-GCA* gene was duplicated, and the total length of the PCGs was 12,378 bp, accounting for 59% of the total genome length of the mitochondria in *R. variegatus*, and the tRNA and rRNA contents were 6.7% and 10.5%, respectively.

**Figure 5:**
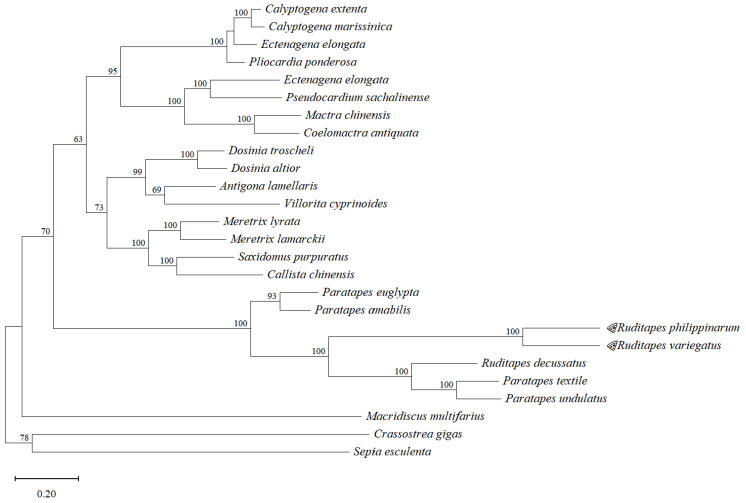
Maximum-likelihood phylogenetic tree constructed for *Ruditapes philippinarum* and *R. variegatus*.

Phylogenetic trees constructed using genome data are more in line with traditional taxonomies based on morphological characters (Figure 5); however, in evolutionary trees constructed based on PCGs, individual species can differ from their traditional taxonomic status. We performed the phylogenetic tree construction through the mitochondrial genome-wide and tandem protein-encoding genomes of *R. philippinarum* and *R. variegatus*. The results showed that the phylogenetic trees constructed using genome-wide associations were consistent with the traditional morphological taxonomies (Figure 5: only the evolutionary trees constructed using the whole genome were shown). Overall, the phylogenetic tree branched into four subclades. The species of Vesicomyidae, Mactridae, and Veneridae clustered into the first large clade; *R. philippinarum* and *R. variegatus* clustered with the species of Veneridae into a second subclade; *Crassostrea gigas* of family Ostreidae clustered with *Sepia esculenta* of family Sepiidae; and *Macridiscus multifarius* of family Veneridae clustered alone as a subclade. As exogenous populations, *Crassostrea gigas* with *Sepia esculenta* together diverged as the outermost branch. On the whole, the representatives of the Veneridae split into three parts. Both the PCGs and the genome-wide constructed evolutionary trees support *R. philippinarum* and *R. variegatus* individually on the developmental tree, and these species are evolutionarily close to the polyclad genus *Paphia*. The phylogenetic tree constructed by this method is consistent with the species’ traditional taxonomic status, which further affirms the accuracy of the evolutionary tree classification.

### 3.2. Comparison of Gene Order in R. philippinarum and R. variegatus

**Figure 6:**
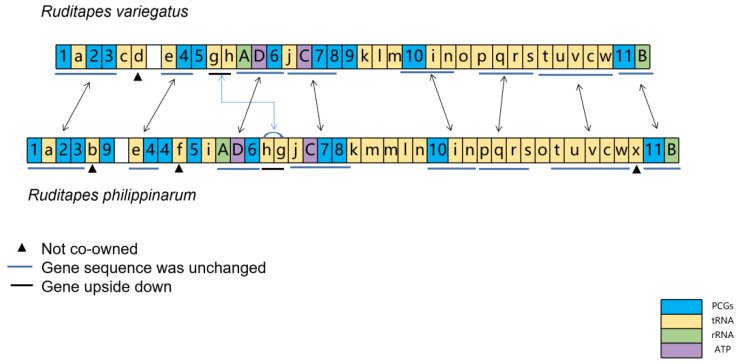
Comparison of mitochondrial DNA gene sequences between *Ruditapes philippinarum* and *R. variegatus*. The lowercase letters a–x respectively denote: trnL-TAG, trnY-ATA, trnC-GCA, trnP-AGG, trnI-GAT, trnP-TGG, trnE-TTC, trnH-GTG, trnN-TTT, trnS-TGA, trnY-GTA, trnD-GTC, trnM-CAT, trnV-TAC, trnG-TCC, trnF-GAA, trnW-TCA, trnR-TCG, trnL-TAA, trnQ-TTG, trnN-GTT, trnT-TGT, trnA-TGC, and trnS-TCT; the numbers 1–11 respectively denote COX1, ND1, ND2, COX2, CYTB, ND4, ND3, ND5, ND4L, ND6, and COX3; capital letters A–D indicate rrnL, ATP8, ATP6, and rrnS, respectively.

The comparative sequence of mitochondrial genes between *R. philippinarum* and *R. variegatus* shows high gene sequence similarity (Figure 6); their mitochondrial DNA share eight identical gene clusters, namely 1-a-2-3, e-4, A-D-6, C-7-8, 10-i-n, p-q-r-s, t-u-v-c-w, and 11-B (short blue lines). Moreover, the sequential order of these eight gene clusters is consistent in the mitochondrial genomes of *R. philippinarum* and *R. variegatus*, and no gene separation occurred during evolution. The highly similar order of mitochondrial genes indicates that *R. philippinarum* and *R. variegatus* are phylogenetically close, affirming the result that *R. philippinarum* and *R. variegatus* have phylogenetic proximity. Second, the gene cluster g-h of *R. philippinarum* and *R. variegatus* are mutually reversed in the arrangement; the gene *trnP-AGG* in *R. variegatus* was not found in *R. philippinarum*; and the genes *trnY-ATA*, *trnP-TGG*, and *trnS-TCT* in *R. philippinarum* mitochondria were absent in *R. variegatus*.

### 3.3. Analysis of Codon Usage Patterns of the PCGs

Table 7 presents basic information about the correlation between the initiation codons and gene lengths of the mitochondrial PCGs of *R. philippinarum* and *R. variegatus*. The genome of *R. philippinarum* contains 14 PCGs, all located on the heavy chain, and the gene *COX2* contains two replicates. The start codons are ATN, GTG or GTG, where gene *ND3* takes GTG as the start codon, and genes *ND5* and *ND6* take TTG as the start codon, and the stop codon is the typical ATG or TAA, where ATG is the start methionine codon. *Ruditapes variegatus* contains 13 PCGs, all of which are likewise located on the heavy chain; the start codons are GTG, ATG, TTG or ATT, with most using ATG as the start codon, but with genes *COX1* and *ND5* using GTG as the start codon, genes *COX2* and *ATP8* using TTG as the start codon, and with ATG and TAA as the stop codons.

**Figure 7:**
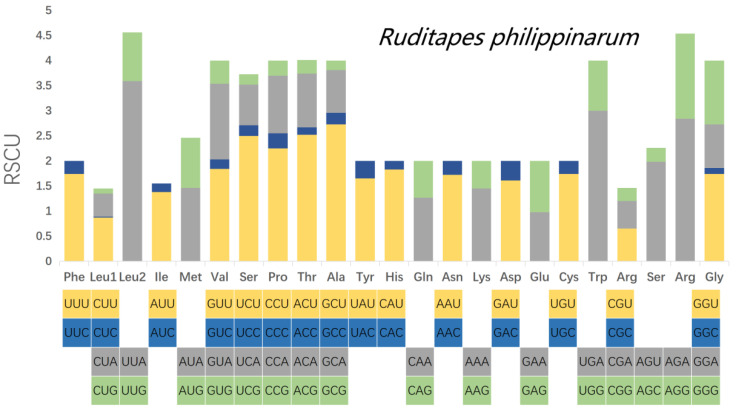
Relative synonymous codon usage (RSCU) in the mitochondrial genome of *Ruditapes philippinarum*.

**Figure 8:**
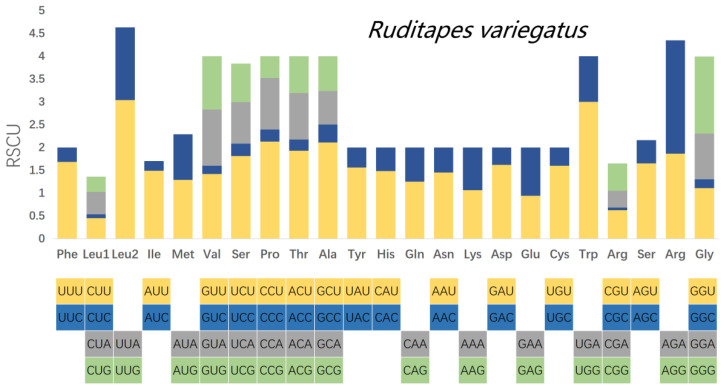
Relative synonymous codon usage (RSCU) in the mitochondrial genome of *Ruditapes variegatus*.

Relative synonymous codon usage (RSCU) was calculated for the mitochondrial PCGs of *R. philippinarum* and *R. variegatus*, as it is generally believed that similar species will have similar codon usage frequencies. Comparative analysis of RSCU values for white matter encoding genes in *R. philippinarum* and *R. variegatus* are summarized in Table 8 and Figure 7 and Figure 8. Excluding the stop codons, the PCGs of *R. philippinarum* consisted of 5189 codons, and the PCGs of *R. variegatus* consisted of 4081 codons. Both 2-fold and 4-fold degenerates have significant AT bias, as shown in Figure 7 and Figure 8 (orange and gray). Codon usage analysis indicated that leucine (*Leu*) and arginine (*Arg*) are the most commonly used amino acid residues, and isoleucine (*IIe*) is the least used amino acid residue.

### 3.4. Codon-Based Analysis of Selective Pressure

**Figure 9:**
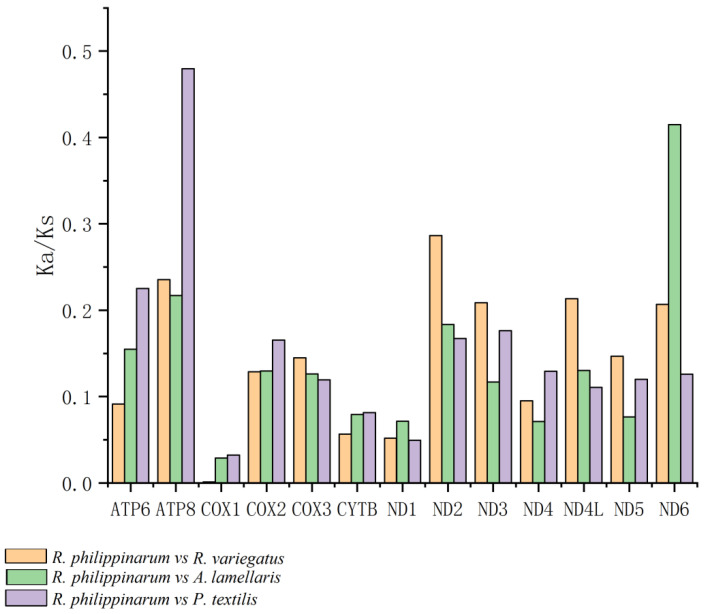
Values of the Ka/Ks ratio in 13 protein-coding genes of *Ruditapes philippinarum* in relation to those of *R. variegatus*, *Antigona lamellaris*, and *Paratapes textilis*.

**Figure 10:**
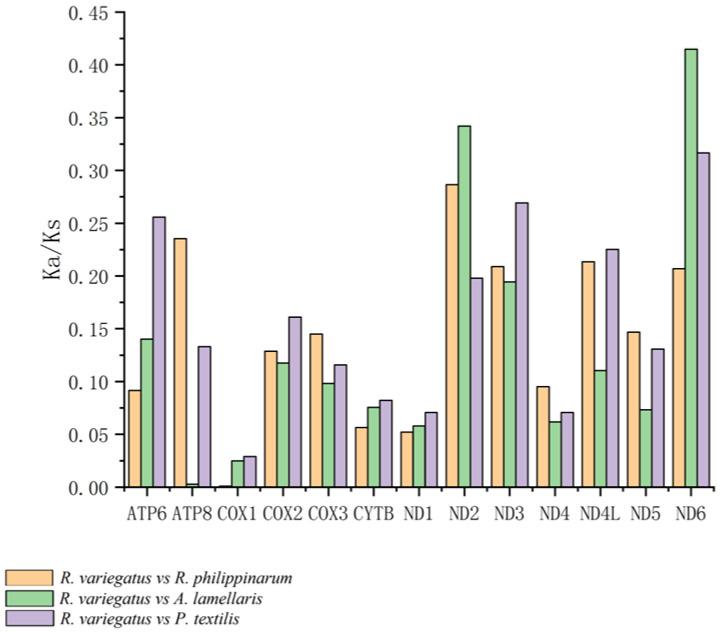
Values of the Ka/Ks ratio in 13 protein-coding genes of *Ruditapes variegatus* in relation to those of *R. philippinarum*, *Antigona lamellaris*, and *Paratapes textilis*.

Values of the Ka/Ks ratio for the pairwise alignments of the 13 homologous PCGs of *Ruditapes philippinarum*, *Antigona lamellaris*, and *Paratapes textilis* are shown in Figure 9. The three genes with Ka/Ks values greater than 0.28 were *ATP8* (0.479: *R. philippinarum* vs. *P. textilis*), *ND6* (0.415: *R. philippinarum* vs. *A. lamellaris*), and *ND2* (0.286: *R. philippinarum* vs. *P. textilis*). Figure 10 shows the Ka/Ks values for the alignment of 13 homologous PCGs between *R. variegatus* and *A. lamellaris*, and between *R. variegatus* and *P. textilis*. The four genes with Ka/Ks values greater than 0.28 were *ND6* (0.415: *R. variegatus* vs. *A. lamellaris*), *ND2* (0.342: *R. variegatus* vs. *A. lamellaris*), *ND6* (0.316: *R. variegatus* vs. *P. textilis*), and *ND2* (0.286: *R. variegatus* vs. *R. philippinarum*).

The Ka/Ks ratios of the 13 PCGs of *R. philippinarum* and *R. variegatus* versus those of *A. lamellaris* or *P. textilis* all yielded values of <1. Moreover, the Ka/Ks values of the genes *COX1*, *COX2*, *COX3*, *CYTB*, *ND1*, and *ND4* are small and have similar values. The alignment of *R. philippinarum* to the *ATP8* gene of *P. textilis* revealed that the Ka/Ks values of the *ATP8* gene of *R. philippinarum* (0.479: *R. philippinarum* vs. *P. textilis*) were significantly greater than those of the *ATP8* gene of *R. variegatus* (0.133: *R. variegatus* vs. *P. textilis*). With *A. lamellaris* as the alignment target, the Ka/Ks value of the *ATP8* gene of *R. variegatus* is 0.003 (*R. variegatus* vs. *A. lamellaris*), which is close to 0, while that of the *ATP8* gene of *R. philippinarum* is 0.217 (*R. philippinarum* vs. *A. lamellaris*), and the difference was significant.

In contrast, the comparison of the Ka/Ks values between *R. philippinarum* and *R. variegatus* versus *A. lamellaris* or *P. textilis* revealed that both have the largest number of relative nonsynonymous substitutions in genes *ND6* and *ND2*. The Ka/Ks values were 0.415 (*R. philippinarum* vs. *A. lamellaris*), 0.415 (*R. variegatus* vs. *A. lamellaris*), 0.316 (*R. variegatus* vs. *P. textilis*), and 0.342 (*R. variegatus* vs. *A. lamellaris*), which are significantly higher than for the other groups; this indicates that the nonsynonymous substitution rate of genes *ND6* and *ND2* has been large in the course of evolution (i.e., variations in genes *ND6* and *ND2* are large). Furthermore, the Ka/Ks values for gene *ND2* aligned to *A. lamellaris*, and the Ka/Ks values for genes *ND4L* and *ND6* aligned to *P. textilis* and *R. variegatus* were greater than those for *R. philippinarum*.

## 4. Discussion

### 4.1. Expression of PCGs under Selective Pressure

Mitochondrial DNA has high mutation rates; numerous nucleotide substitutions may also accumulate quickly between diverging species in the short term, thus facilitating screening for genetic changes over a relatively short period in studies of molecular phylogenetics and evolution. This in turn allows comparisons of differences between homologous genetic loci in different species to determine their evolutionary relatedness. Calculating the evolutionary selection pressure (values of the Ka/Ks ratio) of mitochondrial PCGs in *Ruditapes philippinarum* and *R. variegatus* was significant for reconstructing a phylogeny and understanding the protein-coding sequence [30]. Selection pressure analysis makes it possible to detect mutated genes in species during their gradual adaptation to the environment. The Ka/Ks ratio represents the rates of nonsynonymous and synonymous substitutions between two species. It is generally accepted that PCGs are under positive selection (i.e., mutations are beneficial to the organism) when Ka/Ks > 1, under purifying selection (mutations are harmful to the organism) when Ka/Ks < 1, or under no selection (mutations have no effect) when Ka/Ks = 1. Therefore, by calculating the rates of nonsynonymous and synonymous substitutions of the mitochondrial PCGs for *R. philippinarum* and *R. variegatus*, it is evident that natural selection can act in the short term to change genes during evolution, which thus requires us to determine whether these changes are related to environmental pressure on the organism.

As mentioned, two other species of Veneridae, *Antigona lamellaris* and *Paratapes textilis*, were selected for comparison with *R. philippinarum* and *R. variegatus* for relative synonymous substitution rates at the codon level. Hence, comparison of the calculated values of Ka/Ks could signify the degree of recent variation and enrichment in the mitochondria among these members of the same family. In calculating the Ka/Ks values for the PCGs of *R. philippinarum* and *R. variegatus*, respectively, the same two species (*Antigona lamellaris* and *Paratapes textilis*) were used for the alignment. Therefore, the Ka/Ks ratio served to determine the extent to which the environment caused nucleotide substitutions in mitochondrial genes during evolution through observation of differential gene expression in the mitochondria of the two species.

In this study, all genes had a Ka/Ks value of <1, indicating that the mutation sites between *R. philippinarum* and *R. variegatus* did not reach the level of positive selection pressure when aligned with the species *A. lamellaris* and *P. textilis*, which showed values that manifest as purifying selection—that is, no deleterious genetic mutations remain. The mitochondrial *COX I* gene had the smallest Ka/Ks value and bears the largest purifying selection pressure, indicating high similarity of this gene between *R. philippinarum* and *R. variegatus*. The Ka/Ks value of the mitochondrial *ATP8* gene in *R. philippinarum* was significantly greater than that of *R. variegatus* (Figure 9 and Figure 10), meaning that nonsynonymous substitution of *APT8* in the genome of *R. philippinarum* represents environmental adaptation because it is preserved owing to less environmental pressure. Compared with the *ATP8* gene in *P. textilis*, the Ka/Ks value of *ATP8* in *R. philippinarum* (0.479: *R. philippinarum* vs. *P. textilis*) was significantly greater than the *ATP8* gene in *R. variegatus* (0.133: *R. variegatus* vs. *P. textilis*); and compared with the *ATP8* gene in *A. lamellaris*, the Ka/Ks value of *R. variegatus* is practically 0 (0.003: *R. variegatus* vs. *A. lamellaris*), while the *ATP8* gene in *R. philippinarum* is 0.217 (*R. philippinarum* vs. *A. lamellaris*). In addition, the mitochondrial genes *ND2* and *ND6* in *R. variegatus* were under greater selective pressure during evolution than those genes in *R. philippinarum*, and the nonsynonymous substitution rate of *ND2* and *ND6* in *R. variegatus* was greater than that of *R. philippinarum*, and more mutations were retained. This reveals that mutations formed by the mitochondrial gene *ATP8* of *R. philippinarum* and the mitochondrial genes *ND6* and *ND2* of *R. variegatus* have benefited these species’ adaption to their environments, and that these genes are under greater environmental pressure during genetic changes than other mitochondrial PCGs, which in *R. philippinarum* is mainly manifested on the gene *ATP8*, and in *R. variegatus* on genes *ND6* and *ND2*.

The *ATP8* gene is characterized by rapid adaptation changes, high interspecific mutation rates, and low intraspecific mutation rates, which can produce substantial diversity in a short period. The *ATP8* gene tends to be particularly short, with only 100–200 bp in most animals, thus it can be difficult to detect; the *ATP8* gene in both *R. philippinarum* and *R. variegatus* is 120 bp. Mietanka et al. found that *ATP8* was crucial to the adaptive evolution of marine mussels [31]. Wu et al. likewise found that the *ATP6* and *ATP8* genes exhibited relatively fast rates of evolution [32]. Uddin et al. analyzed the compositional characteristics and codon usage bias of the *ATP6* and *ATP8* genes in fish, birds, and mammals; they discovered an uneven distribution of total bases at the third codon position in these genes in distinct species, and that the GC content of the *ATP8* gene was highest in fish when compared with that in birds and mammals [33]. Nevertheless, the mitochondrial genes of *R. philippinarum* and *R. variegatus* were found to have a high AT bias, which may be due to fish being vertebrates, whereas mollusks differ in their mitochondrial energy metabolism. In an analysis of the mitochondrial genes of Eriosomatinae (woolly aphids), Lee et al. found that use of the *ATP8* gene in DNA barcodes improved the accuracy of species identification in that and other insect taxa [34]. The finding that *ATP8* exhibited lower intraspecific and higher interspecific genetic differences than *COI* is consistent with the large difference in relative codon usage of the mitochondrial *ATP8* gene between *R. philippinarum* and *R. variegatus* found in this study, because patterns of codon usage are thought to be well conserved during evolution [30].

Liu et al. described how the deletion of the *ATP8* gene in the mitochondrial genome of marine shellfish could have special significance for their evolution and gene function [26]. Those authors analyzed the mitochondrial genomes of 16 bivalve species and found that *ATP8* was absent in all 14 marine shellfish examined, except for *Hiatella arctica*, but that the *ATP6* gene was present; however, two freshwater bivalve species possessed the *ATP8* gene. Liu et al. also speculated that because most marine shellfish were benthic and inhabit shallow rocky or sandy substrates in waters with low temperatures, they had low energy metabolism, resulting in degradation of the *ATP8* gene coding function and the loss of gene segments [26]. In contrast, freshwater shellfish have higher levels of energy metabolism as they live in terrestrial freshwater waterbodies with generally higher temperatures. This is similar to the differential expression of the *ATP8* gene in this study. Furthermore, Bernhard et al. noted the loss of *ATP8* within flatworms and that this loss was restricted to the parasitic taxon Neodermata; they concluded that the deletion of the gene in parasitic flatworms, which did not require active temperature regulation, clearly indicateed that *ATP8* was associated with temperature-regulated metabolism [35]. In summary, *R. philippinarum* has a higher nonsynonymous replacement rate than *R. variegatus* for gene *ATP8*. *Ruditapes philippinarum* can better tolerate low temperatures compared with *R. variegatus*; accordingly, *R. philippinarum* is distributed along the north and south coasts of China, while *R. variegatus* is only distributed south of Pingtan (119°97′ E, 24°60′ N) in Fujian Province.

Lee et al. found that the *ATP8* gene evolveed faster than the *COI* gene if the genetic difference between individuals was large enough. However, if it was less than the threshold (i.e., *COI* distance is 1%) then *ATP8* evolveed slower than *COI* [34]. Moreover, the genetic differentiation model of *ATP8* is consistent with divisions in existing classification systems. For instance, Yi et al. investigated genetic diversity using 126 samples from 14 Bactrian camel populations from different regions to distinguish wild haplogroups (H1) and domestic haplogroups (H2–H16) through the mitochondrial genes *ATP8* and *ATP6* [36]. Additionally, the specific regulatory function of gene *ATP8* has not been widely studied, and only *Smt1p* is found to regulate the translation of mitochondrial *ATP8/ATP6* mRNA, as the expression of the mitochondrial-encoded *ATP6* and *ATP8* genes is translationally regulated by F1-ATPase. To further determine whether *ATP8* is a key regulation gene for the temperature adaptability difference between *R. philippinarum* and *R. variegatus*, in-depth research examining transcriptional expression in these two shellfish is needed.

The mitochondrial gene *ND2* plays a key role in controlling the production of mitochondrial reactive oxygen species. Yu et al. found that *mt-ND2* was widely expressed in various tissues and its expression was influenced by dietary fat type and age [37]. Moreover, *mt-ND2* could be considered as a candidate gene associated with adaptation to high-altitude environments [38]. The gene *ND2* has a fast evolutionary rate; this difference in evolutionary rate does not result in significant incongruence between phylogenies derived from the two gene regions independently [39]. However, the gene *ND6* was found to have little genetic variation at the intraspecific level, but large interspecific genetic variation [40]. This is consistent with the results of our study, in which the mitochondrial genes *ND6* and *ND2* differed most between the two species. In this study, *R. variegatus* showed less selective pressure in *ND2* and *ND6* compared with *R. philippinarum*, which had a wide temperature range and relatively wide distribution, whereas *R. variegatus* had a restricted distribution. Based on the present results, it can be inferred that the mitochondrial genes *ND2* and *ND6* are essential in the determination of interspecific differences.

### 4.2. Comparison of Gene Order

Comparison of mitochondrial gene arrangements has become a powerful means to infer evolutionary relationships in animals. The rearrangement of mitochondrial gene order was often considered unique (Boore, 1999), as even two evolutionarily close species might differ substantially in their mitochondrial genomes [41]. The order of gene clusters was similar in *R. philippinarum* and *R. variegatus*, as manifested mainly in eight gene clusters arranged in the same order. The differences were as follows: both species have their own unique genes (noncommon genes); tRNA *trnP-AGG* is present in the mitochondrial genome of *R. variegatus*, but not *R. philippinarum*; and *trnY-ATA*, *trnP-TGG*, and *trnS-TCT* were contained in the mitochondrial genome of *R. philippinarum*, but were absent in *R. variegatus*. Phylogenetic analysis showed that *R. philippinarum* and *R. variegatus* comprise a sister group. The order of mitochondrial genes between *R. philippinarum* and *R. variegatus* was compared in accordance with the tandem duplication–random loss (TDRL) model. The results show a relationship between k-m-m-l-n in *R. philippinarum*, and between K-l-m in *R. variegatus*; this allows speculation that tandem duplication followed by random loss occurs in k-l-m of *R. variegatus*, and in k-m-m-l-n of *R. philippinarum* [12,42,43].

To date, the TDRL model, as a stochastic approximation method, is the most widely accepted hypothesis used to explain the mechanism of mitochondrial gene rearrangement, assuming that the order of gene rearrangement occurs by tandem duplication and a new gene order is generated by random loss of certain repetitive genes or gene clusters [42,44]. A certain nonfunctional gene or gene cluster in a repeat gene is randomly lost or deleted to form a new gene arrangement sequence under natural selection [42]. Previous authors suggested that mitochondrial gene rearrangements could be used as auxiliary molecular markers for phylogenetic relationships because the mitochondrial gene order of close species was relatively stable [45,46,47]. Du et al. asserted that the order of mitochondrial gene arrangement in animals remains unchanged over long periods, although during rapid evolution of animal mitochondrial genome sequences, there is the theoretical possibility of large genome rearrangements [47,48]. The rearrangement of mitochondrial gene sequences has also been found in humans. Poulton et al. detected a family of mtDNA rearrangements in patients with mtDNA deletions [49], which suggests that rearrangements may be a transient intermediate form and that replicating molecules may be intermediate in deletion formation, as a manner of replication that could be a general mechanism for major mitochondrial DNA rearrangements.

## 5. Conclusions

*Ruditapes philippinarum* is found in both northern and southern coastal areas in China, whereas *R. variegatus* is mainly distributed on the south coast in Pingtan, Fujian Province. These venerids have a similar shell morphology and shell color, thus making them difficult to distinguish. This study determined the species’ whole mitochondrial genomes and compared them with two other venerids of genus *Venus*, also found in China. The results showed that *R. philippinarum* differed from *R. variegatus* in basic mitochondrial genome characteristics, which resolveed a historic confusion in classification of the two species. The phylogenetic analysis found that *R. philippinarum* and *R. variegatus* together formed a small branch on the constructed phylogenetic tree. The order of genes is highly similar in *R. philippinarum* and *R. variegatus*, substantiating that the two are close phylogenetically, although the existence of different coding genes indicates certain differences.

We speculate that as external environmental factors (e.g., geographic area and water temperature) that limit the flow between two genes diminish, and as the genetic distance gradually increases, the two species will gradually evolve. The mitochondrial gene orders in *R. philippinarum* and *R. variegatus* include noncommon genes; we also found that tandem duplication followed by random loss could occur between two of the gene clusters, namely k-l-m in *R. variegatus* and k-m-m-l-n in *R. philippinarum*. The PCGs of *R. philippinarum* and *R. variegatus* are significantly AT skewed. Our analysis of selection pressure shows that the nonsynonymous substitution rate in the *ATP8* gene is significantly higher in *R. philippinarum* than in *R. variegatus*; the differential gene enrichment of the mitochondrial gene *ATP8* is found to be associated with temperature-regulated related metabolism, which aligns with the geographical distribution background of the two species in this study, whereby *R. philippinarum* has a wider range and *R. variegatus* has a narrow geographic distribution. This investigation of differences in the mitochondrial genomes of *R. philippinarum* and *R. variegatus* presents scientific evidence for estimating interspecific phylogeny, molecular germplasm characterization, and crossbreeding.

## Figures and Tables

**Table 1:** Comparison of phenotype traits between *Ruditapes philippinarum* (*n* = 30) and *R. variegatus* (*n* = 30), made from specimens collected on the south coast of China.

Material	Shell	Shell	Shell	Tissue Dry	Meat
	Length	Width	Height	Weight	Condition
*R. variegatus*	32.66 ± 2.07	14.37 ± 1.56	23.67 ± 1.57	0.27 ± 0.05	6.11 ± 1.85
*R. philippinarum*	34.96 ± 1.33	15.28 ± 0.69	23.56 ± 1.03	0.28 ± 0.06	7.30 ± 1.40

**Table 2:** List of 24 mollusk species analyzed to construct a phylogenetic tree for *Ruditapes philippinarum* and *R. variegatus* with the maximum-likelihood method.

Species	Family	bp Length	GenBank Accession No.
*Calyptogena marissinica*	Vesicomyidae	17,374	NC_044766.1
*Calyptogena extenta*	Vesicomyidae	16,106	MF981085.1
*Ectenagena elongata*	Vesicomyidae	16,827	NC_051454.1
*Pliocardia ponderosa*	Vesicomyidae	16,275	MF981084.1
*Pseudocardium sachalinense*	Mactridae	17,978	MG431821.1
*Mactra antiquata*	Mactridae	17,199	JQ423460.1
*Mactra chinensis*	Mactridae	17,285	KJ754823.1
*Lutraria maxima*	Mactridae	17,082	MF784266.1
*Paphia amabilis*	Veneridae	19,629	JF969276.1
*Paratapes textilis*	Veneridae	18,561	JF969277.1
*Paratapes undulatus*	Veneridae	18,154	JF969278.1
*Paphia euglypta*	Veneridae	18,643	GU269271.1
*Macridiscus multifarius*	Veneridae	20,171	NC_045888.1
*Ruditapes decussatus*	Veneridae	18,995	KP089983.1
*Dosinia altior*	Veneridae	17,536	NC_037916.1
*Dosinia troscheli*	Veneridae	17,229	NC_037917.1
*Saxidomus purpurata*	Veneridae	19,637	KP419933.1
*Callista chinensis*	Veneridae	19,703	MT742541.1
*Meretrix lamarckii*	Veneridae	21,209	GU071281.1
*Villorita cyprinoides*	Veneridae	15,880	NC_050989.1
*Meretrix lyrata*	Veneridae	21,625	KC832317.1
*Antigona lamellaris*	Veneridae	17,532	NC_051506.1
*Sepia esculenta*	Sepiidae	16,199	NC_009690.1
*Crassostrea gigas*	Ostreidae	18,224	MZ497416.1

**Table 3:** Gene content of the *Ruditapes philippinarum* genome.

Group of Genes	Gene Name
complex Ⅰ (NADH dehydrogenase)	ND1, ND2, ND3, ND4, ND5, ND6, ND4L
complex Ⅳ (cytochrome c oxidase)	COX1, COX2 (2 copies), COX3
ATP synthase	ATP6, ATP8
other genes	CYTB
transfer RNAs	trnY-ATA, trnY-GTA, trnl-GAT, trnP-TGG, trnN-TTT, trnN-GTT (2 copies), trnH-GTG, trnE-TTC, trnS-TGA, trnS-TCT, trnM-CAT (2 copies), trnM-CAT, trnD-GTC, trnV-TAC (2 copies), trnF-GAA, trnW-TCA, trnR-TCG, trnG-TCC, trnQ-TTG, trnT-GCA, trnA-TGC, trnL-TAG, trnL-TAA
ribosomal RNAs	RrnL, rrnS
origin of replication	D-loop

**Table 4:** Genomic features of *Ruditapes philippinarum*.

*Ruditapes philippinarum*	A%	C%	G%	T%	GC%	Size (bp)	Proportion in Genome (%)
Whole genome	30.2	9.80	20.5	39.6	30.30	22,706	100
Protein-coding genes	27.4	9.9	21.7	41.1	31.57	14,454	63.66
tRNA genes	32.8	11.8	18.2	37.3	29.97	1685	7.42
rRNA genes	35.4	10.4	18.5	35.7	28.87	2054	9.05
D-loop	34.7	12.0	21.6	31.7	33.65	1777	7.83

**Table 5:** Gene content of the *Ruditapes variegatus* genome.

Group of Genes	Gene Name
complex Ⅰ (NADH dehydrogenase)	ND4, ND3, ND5, ND4L, ND6, ND1, ND2
complex Ⅳ (cytochrome c oxidase)	COX2, COX3, COX1
ATP synthase	ATP8, ATP6
other genes	CYTB
transfer RNAs	trnC-GCA (2 copies), trnP-AGG, trnl-GAT, trnE-TTC, trnH-GTG, trnS-TGA, trnY-GAT, trnD-GTC, trnM-CAT, trnN-TTT, trnN-GTT, trnV-TAC, trnG-TCC, trnF-GAA, trnW-TCA, trnR-TCG, trnL-TAA, trnL-TAG, trnQ-TTG, trnT-TGT, trnA-TGC
ribosomal RNAs	rrnL, rrnS
origin of replication	D-loop

**Table 6:** Genomic features of *Ruditapes variegatus*.

*Ruditapes variegatus*	A%	C%	G%	T%	GC%	Size (bp)	Proportion in Genome (%)
Whole genome	28.1	10.7	25.7	35.5	36.4	20,997	100
Protein-coding genes	24.7	10.8	26.2	38.3	37.0	12,378	59.0
tRNA genes	30.9	12.7	22.7	33.7	35.4	1414	6.7
rRNA genes	33.5	11.3	22.1	33.1	33.4	2207	10.5
D-loop	33.4	9.4	27.1	30.1	36.5	3892	18.5

**Table 7:** Genomic organization and expression profile of the mitochondrial protein-coding genes in *Ruditapes philippinarum* and *R. variegatus*.

*Ruditapes philippinarum*					*Ruditapes variegatus*				
Gene Name	Length	Start Codon	Stop Codon	Direction	Gene Name	Length	Start Codon	Stop Codon	Direction
COX1	1719	ATA	TAG	+	COX1	1758	GTG	TAG	+
ND1	930	ATA	TAA	+	ND1	933	ATG	TAG	+
ND2	1035	ATT	TAG	+	ND2	1029	ATG	TAG	+
ND4L	282	ATT	TAA	+	COX2	1416	TTG	TAG	+
COX2	1995	ATC	TAA	+	CYTB	1239	ATT	TAA	+
COX2	1608	ATA	TAA	+	ATP8	120	TTG	TAA	+
CYTB	1224	ATT	TAA	+	ND4	1356	ATG	TAA	+
ATP8	120	ATT	TAG	+	ATP6	735	ATG	TAG	+
ND4	1359	ATG	TAA	+	ND3	405	ATG	TAA	+
ATP6	738	ATA	TAG	+	ND5	1704	GTG	TAA	+
ND3	405	GTG	TAA	+	ND4L	279	ATG	TAG	+
ND5	1665	TTG	TAA	+	ND6	522	ATG	TAG	+
ND6	510	TTG	TAA	+	COX3	882	ATT	TAA	+
COX3	864	ATT	TAA	+					

**Table 8:** Codon number and relative synonymous codon usage (RSCU) in the mitochondrial genomes of *Ruditapes philippinarum* and *R. variegatus*.

*Ruditapes philippinarum*						*Ruditapes variegatus*					
Codon	Count	RSCU	Codon	Count	RSCU	Codon	Count	RSCU	Codon	Count	RSCU
UUU(F)	430	1.74	GCG(A)	9	0.19	UUU(F)	324	1.68	GCG(A)	37	0.76
UUC(F)	63	0.26	UAU(Y)	171	1.65	UUC(F)	61	0.32	UAU(Y)	140	1.56
UUA(L)	430	3.59	UAC(Y)	36	0.35	UUA(L)	265	3.04	UAC(Y)	40	0.44
UUG(L)	116	0.97	CAU(H)	73	1.83	UUG(L)	139	1.59	CAU(H)	46	1.48
CUU(L)	104	0.87	CAC(H)	7	0.17	CUU(L)	39	0.45	CAC(H)	16	0.52
CUC(L)	2	0.02	CAA(Q)	38	1.27	CUC(L)	7	0.08	CAA(Q)	38	1.25
CUA(L)	55	0.46	CAG(Q)	22	0.73	CUA(L)	43	0.49	CAG(Q)	23	0.75
CUG(L)	12	0.1	AAU(N)	153	1.72	CUG(L)	30	0.34	AAU(N)	72	1.45
AUU(I)	239	1.38	AAC(N)	25	0.28	AUU(I)	174	1.49	AAC(N)	27	0.55
AUC(I)	29	0.17	AAA(K)	138	1.45	AUC(I)	25	0.21	AAA(K)	71	1.06
AUA(M)	253	1.46	AAG(K)	53	0.55	AUA(M)	151	1.29	AAG(K)	63	0.94
AUG(M)	79	1	GAU(D)	99	1.61	AUG(M)	98	1	GAU(D)	77	1.62
GUU(V)	238	1.84	GAC(D)	24	0.39	GUU(V)	154	1.42	GAC(D)	18	0.38
GUC(V)	25	0.19	GAA(E)	91	0.98	GUC(V)	20	0.18	GAA(E)	66	0.94
GUA(V)	195	1.51	GAG(E)	95	1.02	GUA(V)	134	1.23	GAG(E)	74	1.06
GUG(V)	59	0.46	UGU(C)	105	1.74	GUG(V)	127	1.17	UGU(C)	88	1.6
UCU(S)	141	2.5	UGC(C)	16	0.26	UCU(S)	68	1.81	UGC(C)	22	0.4
UCC(S)	12	0.21	UGA(W)	65	3	UCC(S)	10	0.27	UGA(W)	39	3
UCA(S)	46	0.81	UGG(W)	72	1	UCA(S)	34	0.91	UGG(W)	65	1
UCG(S)	12	0.21	CGU(R)	35	0.65	UCG(S)	32	0.85	CGU(R)	30	0.62
CCU(P)	82	2.25	CGC(R)	0	0	CCU(P)	66	2.13	CGC(R)	3	0.06
CCC(P)	11	0.3	CGA(R)	30	0.55	CCC(P)	8	0.26	CGA(R)	18	0.37
CCA(P)	42	1.15	CGG(R)	14	0.26	CCA(P)	35	1.13	CGG(R)	29	0.6
CCG(P)	11	0.3	AGU(S)	112	1.98	CCG(P)	15	0.48	AGU(S)	62	1.65
ACU(T)	104	2.52	AGC(S)	16	0.28	ACU(T)	57	1.93	AGC(S)	19	0.51
ACC(T)	6	0.15	AGA(R)	154	2.84	ACC(T)	7	0.24	AGA(R)	90	1.86
ACA(T)	44	1.07	AGG(R)	92	1.7	ACA(T)	30	1.02	AGG(R)	121	2.49
ACG(T)	11	0.27	GGU(G)	180	1.74	ACG(T)	24	0.81	GGU(G)	98	1.11
GCU(A)	129	2.73	GGC(G)	12	0.12	GCU(A)	103	2.11	GGC(G)	17	0.19
GCC(A)	11	0.23	GGA(G)	90	0.87	GCC(A)	19	0.39	GGA(G)	88	1
GCA(A)	40	0.85	GGG(G)	131	1.27	GCA(A)	36	0.74	GGG(G)	149	1.69

## Data Availability

The data that support the findings of this study are all generated by our experiments. The raw data has been uploaded to the NCBI Sequence Read Ar-chive (SRA). The BioProject ID are PRJNA896365 (*R. philippinarum*) and PRJNA896706 (*R. variegatus*). The nucleotide sequences were uploaded to GenBank (https://www.ncbi.nlm.nih.gov/genome), with accession numbers MZ675529 and MZ675530 for *R. philippinarum* and *R. variegatus*. (accessed on 15 November 2022).
